# Industry and workplace characteristics associated with the downloading of a COVID-19 contact tracing app in Japan: a nation-wide cross-sectional study

**DOI:** 10.1186/s12199-021-01016-1

**Published:** 2021-09-21

**Authors:** Tomohiro Ishimaru, Koki Ibayashi, Masako Nagata, Ayako Hino, Seiichiro Tateishi, Mayumi Tsuji, Akira Ogami, Shinya Matsuda, Yoshihisa Fujino, for the CORoNaWork Project, Yoshihisa Fujino, Hajime Ando, Hisashi Eguchi, Kazunori Ikegami, Tomohiro Ishimaru, Arisa Harada, Ayako Hino, Kyoko Kitagawa, Kosuke Mafune, Shinya Matsuda, Ryutaro Matsugaki, Koji Mori, Keiji Muramatsu, Masako Nagata, Tomohisa Nagata, Ning Liu, Akira Ogami, Rie Tanaka, Seiishiro Tateishi, Kei Tokutsu, Mayumi Tsuji

**Affiliations:** 1 Department of Environmental Epidemiology, Institute of Industrial Ecological Sciences, University of Occupational and Environmental Health, Japan, Kitakyushu, Japan; 2 Department of Occupational Health Practice and Management, Institute of Industrial Ecological Sciences, University of Occupational and Environmental Health, Japan, Kitakyushu, Japan; 3 Department of Mental Health, Institute of Industrial Ecological Sciences, University of Occupational and Environmental Health, Japan, Kitakyushu, Japan; 4 Department of Occupational Medicine, School of Medicine, University of Occupational and Environmental Health, Japan, Kitakyushu, Japan; 5 Department of Environmental Health, School of Medicine, University of Occupational and Environmental Health, Japan, Kitakyushu, Japan; 6 Department of Work Systems and Health, Institute of Industrial Ecological Sciences, University of Occupational and Environmental Health, Japan, Kitakyushu, Japan; 7 Department of Preventive Medicine and Community Health, School of Medicine, University of Occupational and Environmental Health, Japan, Kitakyushu, Japan

**Keywords:** Contact tracing, COVID-19, SARS-CoV-2, Smartphone, Worksite

## Abstract

**Background:**

To combat coronavirus disease 2019 (COVID-19), many countries have used contact tracing apps, including Japan’s voluntary-use contact-confirming application (COCOA). The current study aimed to identify industry and workplace characteristics associated with the downloading of this COVID-19 contact tracing app.

**Methods:**

This cross-sectional study of full-time workers used an online survey. Multiple logistic regression analysis was used to evaluate the associations of industry and workplace characteristics with contact tracing app use.

**Results:**

Of the 27,036 participants, 25.1% had downloaded the COCOA. Workers in the public service (adjusted odds ratio [aOR] = 1.29, 95% confidence interval [CI] 1.14–1.45) and information technology (aOR = 1.38, 95% CI 1.20–1.58) industries were more likely to use the app than were those in the manufacturing industry. In contrast, app usage was less common among workers in the retail and wholesale (aOR = 0.87, 95% CI 0.76–0.99) and food/beverage (aOR = 0.81, 95% CI 0.70–0.94) industries, but further adjustment for company size attenuated these associations. Workers at larger companies were more likely to use the app. Compared with permanent employees, the odds of using the app were higher for managers and civil servants but lower for those who were self-employed.

**Conclusions:**

Downloading of COCOA among Japanese workers was insufficient; thus, the mitigating effect of COCOA on the COVID-19 pandemic is considered to be limited. One possible reason for the under-implementation of the contact tracing app in the retail and wholesale and food/beverage industries is small company size, as suggested by the fully adjusted model results. An awareness campaign should be conducted to promote the widespread use of the contact tracing app in these industries.

## Background

A number of countries have used digital contact tracing tools to combat COVID-19 by tracing possible infected cases to prevent the spread of the disease [1, 2]. Japan’s government released a contact tracing app for smartphones—the COCOA—on 19 June 2020 [3]. Downloading the COCOA is not mandatory but it is recommended by authorities. As of 18 March 2021, the COCOA had been downloaded by 20.8% of Japanese citizens (26 million downloads) and used for 2.5% of positive cases (11,513/452,863) [3]. The COCOA uses Bluetooth to collect information on close contacts—those who have been within 1 m of an individual who has installed the app for a duration of at least 15 min in the last 14 days. When a user is infected with COVID-19, they can alert others with whom they have been in close contact through the app. No personal information is stored in the central database. The notified person can then get tested at the nearest COVID-19 testing center.

High utilization is important for contact tracing apps because they are only effective when both those who are infected with COVID-19 and their close contacts have installed and used the app. Contact tracing conducted using apps has been estimated to reduce the number of infected people by 24 to 71% [4]. Various simulations have been conducted to determine how many tracing applications need to be installed for effective control of COVID-19, with varying results (20–90%) [5–7]. In other countries, contact tracing apps, such as TraceTogether in Singapore, have frequently been used, with mandatory installation and use [2]. In contrast, this practice is less frequent in countries where smartphones are not widely used [2]. In terms of using contact tracing apps, migrant workers, persons with low income, and older adults who may find the technology necessary to use the app difficult are all at a disadvantage [8]. The acceptance rate of contact tracing apps has been found to range from 42 to 70% in developed countries [9–11]. Several factors influence this acceptance: trust in the government, health concerns, privacy, battery usage, and concerns about the effectiveness of the app [10].

In Japan, employers have promoted the use the COCOA among workers for effective contact tracing to prevent workplace COVID-19 clusters [12]. Many workers are at risk of COVID-19 infection because of their close contact with colleagues or customers. However, there is little evidence about the associations of industry and workplace characteristics with the downloading of contact tracing apps. Promoting the use of contact tracing apps in the workplace is also important for preventing the spread of clusters from the working-age population to high-risk populations, such as older adults. To develop a strategy for the widespread use of contact tracing apps, it is necessary to identify the industries or workplaces in which such apps are under-implemented. The purpose of the current study was to identify which industry and workplace characteristics were associated with the downloading of a COVID-19 contact tracing app.

## Methods

### Study design and participants

The current study was conducted as part of the Collaborative Online Research on the Novel-coronavirus and Work (CORoNaWork) Project. Details of the study protocol have been published elsewhere [13]. Briefly, the CORoNaWork Project was an online cohort study in Japan. We extracted online self-administered questionnaire data from the baseline dataset, collected 22–26 December 2020, for cross-sectional analysis. Panelists who had registered with an online research company and who were currently working full-time were invited to participate in the survey. Health care workers and caregivers were not invited to participate. We selected 33,087 participants using cluster sampling, stratified by sex, region, and job type. We excluded invalid responses, which left data on 27,036 participants for analysis. Invalid responses (*n* = 6051) were (a) wrong answer to a question intended to identify false responses; (b) short response time (≤ 6 min); (c) low weight (< 30 kg); (d) short height (< 140 cm); and (e) inconsistent answers within a question. When the baseline survey was conducted, the numbers of COVID-19 infections and deaths were much higher than they had been during the first and second epidemic waves in Japan; therefore, Japan was on maximum alert during this third wave. In detail, the number of newly confirmed cases peaked at 0.5 per 100,000 population during the first wave (9 April 2020), 1.2 per 100,000 population in the second wave (30 July 2020), and 6.2 per 100,000 population during the third wave (7 January 2021) [14]. This study was approved by the Ethics Committee of the University of Occupational and Environmental Health, Japan (R2-079).

### Outcome

The outcome variable, the downloading of the contact tracing app, was assessed with the question “Have you downloaded COCOA?” The response options were “yes” and “no.”

### Explanatory variables

Three variables were used to assess industry and workplace characteristics: type of industry, company size, and occupation. Type of industry followed the Japan Standard Industrial Classification, with industries accounting for less than 3% of the sample categorized as “other” [15]. Company size was classified as < 10, 10–49, 50–999, or ≥ 1000 employees. Each participant reported their occupation as *permanent employee*, *manager*, *civil servant*, *dispatched or contract worker*, *self-employed*, or *other*.

### Covariates

Covariates comprised (a) demographic characteristics; (b) health behavior; and (c) risk perception. The demographic variables included sex, age (20–39, 40–49, 50–59, and 60–65 years), marital status (single; divorced or widowed; and married), education (junior high or high school; vocational school or college; and university or graduate school), and annual household income (< 2,000,000, 2,000,000–3,999,999, 4,000,000–7,999,999, and ≥ 8,000,000 JPY). The health behavior variables were smoking (never, past, and current) and alcohol intake (never, occasionally, and regularly). Risk perception was assessed using a single question on having anxiety about contracting COVID-19, with response options of “yes” and “no.”

### Data analysis

Univariate and multiple logistic regression analyses were used to evaluate the associations of occupational factors with the downloading of the contact tracing app. We assessed three occupational factors as explanatory variables: type of industry, company size, and occupation. Three models were evaluated. Model 1 was adjusted for sex and age. Model 2 was adjusted for sex, age, marital status, education, annual household income, smoking, alcohol intake, and anxiety about contracting COVID-19. Model 3 was additionally adjusted for other variables, including type of industry and company size. We did not include the occupation variables in Model 3 because of the high correlations between the occupation of civil servant and the public service industry and between self-employed occupation and company size of 1–9 employees. All *P* values were two-sided, and statistical significance was set at *P* < 0.05. We used Stata/SE 16.1 (StataCorp, College Station, TX, USA) for all analyses.

## Results

Table 1 shows the general characteristics of the study participants. Of the 27,036 participants, 6786 (25.1%) reported having downloaded the COCOA. Approximately half of the participants were women (48.9%), and about half were married (55.6%). The most frequently observed category for annual household income was 4–8 million JPY (44.1%; USD 1 = JPY 106.78 as of 2020) [16]. Excluding the “other” industry category, manufacturing accounted for the largest percentage of the sample (17.0%), followed by medical and welfare (16.6%) and public service (7.0%). Establishments with 50–999 employees (35.9%) and permanent employee (46.5%) were the most frequently observed company size and occupation categories, respectively.

**Table 1:** General characteristics of the study participants

	Total *N* = 27,036	COVID-19 contact tracing app users *n* = 6786 (25.1%)	
	*n* (%)	*n* (%)	Download rate (%)
Sex			
Female	13,222 (48.9)	3181 (46.9)	24.1
Male	13,814 (51.1)	3605 (53.1)	26.1
Age			
20–39 years	6763 (25.0)	1669 (24.6)	24.7
40–49 years	8011 (29.6)	1886 (27.8)	23.5
50–59 years	9012 (33.4)	2359 (34.8)	26.2
60–65 years	3250 (12.0)	872 (12.8)	26.8
Marital status			
Single	9164 (33.9)	1991 (29.4)	21.7
Divorced or widowed	2843 (10.5)	722 (10.6)	25.4
Married	15,029 (55.6)	4073 (60.0)	27.1
Education			
Junior high or high school	7321 (27.1)	1647 (24.3)	22.5
Vocational school or college	6544 (24.2)	1552 (22.9)	23.7
University or graduate school	13,171 (48.7)	3587 (52.8)	27.2
Annual household income			
< 2,000,000 Japanese yen	1709 (6.3)	277 (4.1)	16.2
2,000,000–3,999,999 Japanese yen	5548 (20.5)	1188 (17.5)	21.4
4,000,000–7,999,999 Japanese yen	11,927 (44.1)	2925 (43.1)	24.5
≥ 8,000,000 Japanese yen	7852 (29.1)	2396 (35.3)	30.5
Smoking			
Non-smoking	14,587 (54.0)	3495 (51.5)	24.0
Past smoking	5445 (20.1)	1551 (22.9)	28.5
Current smoking	7004 (25.9)	1740 (25.6)	24.8
Alcohol intake			
Never	11,472 (42.4)	2699 (39.8)	23.5
Occasionally	4547 (16.8)	1186 (17.5)	26.1
Regularly	11,017 (40.8)	2901 (42.7)	26.3
Anxiety about contracting COVID-19			
Yes	20,017 (74.0)	5737 (84.5)	28.7
No	7019 (26.0)	1049 (15.5)	14.9
Industry			
Manufacturing	4591 (17.0)	1165 (17.2)	25.4
Public service	1891 (7.0)	614 (9.0)	32.5
Information technology	1331 (4.9)	429 (6.3)	32.2
Retail and wholesale	1797 (6.6)	394 (5.8)	21.9
Food/beverage	1319 (4.9)	269 (4.0)	20.4
Medical and welfare	4500 (16.6)	1071 (15.8)	23.8
Finance	1226 (4.5)	364 (5.4)	29.7
Construction	944 (3.5)	240 (3.5)	25.4
Other	9437 (34.9)	2240 (33.0)	23.7
Company size			
1–9 employees	6165 (22.8)	1272 (18.7)	20.6
10–49 employees	4390 (16.2)	957 (14.1)	21.8
50–999 employees	9703 (35.9)	2491 (36.7)	25.7
≥ 1000 employees	6778 (25.1)	2066 (30.4)	30.5
Occupation			
Permanent employee	12,575 (46.5)	3006 (44.3)	23.9
Manager	3403 (12.6)	1160 (17.1)	34.1
Civil servant	2810 (10.4)	840 (12.4)	29.9
Dispatched or contact worker	2894 (10.7)	671 (9.9)	23.2
Self-employed	2819 (10.4)	525 (7.7)	18.6
Other	2535 (9.4)	584 (8.6)	23.0

*COVID-19* coronavirus disease 2019

Table 2 shows the associations of industry and workplace characteristics with the downloading of the COVID-19 contact tracing app. Participants in the public service (adjusted odds ratio [aOR] = 1.29, 95% *c*onfidence interval [CI] 1.14–1.45, Model 3) and information technology (aOR = 1.38, 95% CI 1.20–1.58, Model 3) industries were more likely to use the app than were those in the manufacturing industry. In contrast, app usage was less common among participants working in the retail and wholesale and food/beverage industries, and adjusting for demographic, health behavior, and risk perception variables did not alter these associations remarkably (retail and wholesale: aOR = 0.87, 95% CI 0.76–0.99; food/beverage: aOR = 0.81, 95% CI 0.70–0.94; Model 2); however, after further adjustment for company size in Model 3, these industry types showed no significant difference from the manufacturing industry.

**Table 2:** Associations between occupational factors and the downloading of the COVID-19 contact tracing app

	Model 1			Model 2			Model 3		
	OR	(95% CI)	***P*** value	OR	(95% CI)	***P*** value	OR	(95% CI)	***P***-value
Industry									
Manufacturing	1.00	–	–	1.00	–	–	1.00	–	–
Public service	1.41	(1.25–1.58)	< 0.001	1.32	(1.17–1.49)	< 0.001	1.29	(1.14–1.45)	< 0.001
Information technology	1.39	(1.21–1.58)	< 0.001	1.36	(1.18–1.55)	< 0.001	1.38	(1.20–1.58)	< 0.001
Retail and wholesale	0.83	(0.73–0.94)	0.004	0.87	(0.76–0.99)	0.034	0.92	(0.81–1.05)	0.223
Food/beverage	0.76	(0.66–0.89)	< 0.001	0.81	(0.70–0.94)	0.007	0.89	(0.76–1.03)	0.125
Medical and welfare	0.94	(0.85–1.03)	0.201	0.86	(0.78–0.94)	0.004	0.91	(0.82–1.01)	0.068
Finance	1.26	(1.09–1.45)	0.001	1.19	(1.03–1.38)	0.016	1.15	(0.99–1.32)	0.061
Construction	1.00	(0.85–1.18)	0.999	0.98	(0.84–1.16)	0.845	1.10	(0.93–1.30)	0.271
Other	0.92	(0.84–0.99)	0.034	0.94	(0.87–1.03)	0.175	1.01	(0.93–1.10)	0.807
Company size									
1–9 employees	1.00	–	–	1.00	–	–	1.00	–	–
10–49 employees	1.09	(0.99–1.20)	0.063	1.04	(0.94–1.14)	0.453	1.03	(0.97–1.11)	0.329
50–999 employees	1.36	(1.25–1.46)	< 0.001	1.24	(1.14–1.34)	< 0.001	1.22	(1.13–1.33)	< 0.001
≥ 1000 employees	1.71	(1.57–1.85)	< 0.001	1.52	(1.39–1.65)	< 0.001	1.44	(1.32–1.57)	< 0.001
Occupation*									
Permanent employee	1.00	–	–	1.00	–	–			
Manager	1.62	(1.48–1.76)	< 0.001	1.40	(1.28–1.53)	< 0.001			
Civil servant	1.34	(1.23–1.47)	< 0.001	1.22	(1.11–1.34)	< 0.001			
Dispatched or contact worker	0.95	(0.86–1.04)	0.277	1.02	(0.92–1.12)	0.769			
Self-employed	0.72	(0.64–0.80)	< 0.001	0.76	(0.69–0.85)	< 0.001			
Other	0.95	(0.86–1.05)	0.290	0.89	(0.80–0.99)	0.033			

*COVID-19* coronavirus disease 2019, *OR* odds ratio, *CI* confidence interval

Model 1: Adjusted for sex and age

Model 2: Adjusted for sex, age, income, marital status, education, smoking, alcohol intake, and anxiety about contracting COVID-19

Model 3: Adjusted for sex, age, income, marital status, education, smoking, alcohol intake, anxiety about contracting COVID-19, type of industry, and company size

*The occupation variables were not included in Model 3 because of the high correlations between the occupation of civil servant and the public service industry and between self-employed occupation and company size of 1–9 employees

Participants in larger companies were more likely to use the app (50–999 employees: aOR = 1.21, 95% CI 1.12–1.32; ≥ 1000 employees: aOR = 1.37, 95% CI 1.24–1.52; Model 3). Regarding occupation, compared with permanent employees, the odds of app usage were higher for managers (aOR = 1.40, 95% CI 1.28–1.53, Model 2) and civil servants (aOR = 1.22, 95% CI 1.11–1.34, Model 2) but lower for those who were self-employed (aOR = 0.76; 95% CI 0.69–0.85, Model 2).

## Discussion

The current study identified that working in the public service sector, in the information technology industry, or for companies with 50 or more employees, as well as holding the position of manager or civil servant, were factors facilitating the downloading of the contact tracing app—over 30% of each of these groups had downloaded the app. In contrast, working in the retail and wholesale and food/beverage industries and being self-employed were associated with a lower likelihood of using the app—around 20% of these groups had downloaded the app. These findings give insight needed for the development of a strategy for the widespread use of COVID-19 contact tracing apps.

In this large-scale survey, we found that the downloading of COCOA among Japanese workers remains low (25.1%). The current utilization rate is slightly higher than 20%; this low rate is considered to have a limited mitigating effect on the COVID-19 pandemic [7]. A similar situation was observed in Europe with voluntary installation and use [17]. As countermeasures, many countries encourage users to download and use the tracing app by providing additional functions that users feel are helpful or interesting. For example, the United Kingdom offers a service in which users can receive notifications of COVID-19 infection risk levels for a particular region via the tracing app [17]. Our finding suggests that similar measures to increase the utilization rate of COCOA are necessary in Japan.

An important finding of this study was that the contact tracing app was under-implemented in the retail and wholesale and food/beverage industries. This finding did not change substantially after adjusting for demographic, health behavior, and risk perception variables. However, further adjustment for company size attenuated these associations. One possible reason for this change in results is that most workplaces in these types of industries are small-scale, which may be the cause of insufficient downloading of the app. This idea is consistent with a previous study showing that small companies were relatively unlikely to implement workplace measures against COVID-19 [18]. This may be because of the limited opportunities to obtain information and the lack of human, time, and financial resources to take countermeasures against health threats at small companies [19]. However, workers in these industries have particularly a strong need to use the contact tracing app because they are in contact with an unspecified number of people, such as in restaurants and bars, where customers eat and drink without masks [20]. An awareness campaign should be conducted for the retail and wholesale and food/beverage industries to promote the use of the contact tracing app [6].

We found that the contact tracing app was frequently used by civil servants and managers. This finding implies that civil servants and managers might follow requests to use the app made through organized channels because workers were encouraged to use the COCOA by the government and by employer associations [12]. A previous study conducted in Japan reported that the public service sector most frequently received announcements regarding measures to prevent COVID-19 in the early stage of the pandemic [21]—this also suggests that the public service sector provides COVID-19 advisory information to civil servants. These results indicate that app uptake may increase when workers, including managers, receive messages through organized channels, even if the requests are voluntary. For non-managers, a sharing function allowing users to invite coworkers and friends to download the app may increase the rate of installation of the app [10].

Workers in the information technology industry, such as system engineers, have the advantage of expertise in digital technology and know how to use smartphone apps. The current study revealed that information technology industry workers used the contact tracing app more frequently, compared with workers in other industries. This finding is in line with a previous study showing that high information technology literacy eliminated technical obstacles to using contact tracing apps [22]. In addition, the information technology industry actively implements workplace measures against COVID-19, such as teleworking, and these practices may also relate to the finding [21]. These results suggest that a technical support service for those who have difficulty using the contact tracing app may improve app uptake.

This study has several limitations. First, the study recruited panelists who registered with an online research company. Therefore, the participants may not represent general workers. For example, online panelists may be particularly willing to use online tools or more familiar than others with using apps. However, the estimate of 20.8% using national data on the COCOA download rate and the result of 25.1% in this study were nearly the same, which indicates that selection bias is not a serious issue [3]. Second, because this survey did not target health care workers or caregivers, respondents who stated that they worked in the medical and welfare industry were considered non-medical professionals working in health-related facilities (e.g., medical clerk, cleaning staff, security and food services). Therefore, the results for the medical and welfare industry are not applicable to health care workers or caregivers. Despite these limitations, to the best of our knowledge, this study represents the first study in Japan to examine current downloading of the COCOA with a large sample.

## Conclusion

In the present study, we evaluated the associations of industry and workplace characteristics with downloading of a COVID-19 contact tracing app in a large-scale online survey of Japanese workers. We found that the rate of downloading COCOA among Japanese workers was 25.1%; the mitigating effect of COCOA on the COVID-19 pandemic is considered to be limited. Those working in the public service sector or in information technology, as well as managers, were frequently found to use the contact tracing app, whereas those working in the retail and wholesale and food/beverage industries were less likely to use it. One possible reason for the under-implementation of the contact tracing app in the retail and wholesale and food/beverage industries may be the small size of companies in these types of industries. An awareness campaign should be conducted for workers in these industries to promote the widespread use of the contact tracing app to help these workers trace possible infected cases, preventing the spread of the disease.

## Data Availability

Not applicable.

## Abbreviations

COCOA: Contact-confirming application
COVID-19: Coronavirus disease 2019
